# Modified Bacterial Cellulose Dressings to Treat Inflammatory Wounds

**DOI:** 10.3390/nano10122508

**Published:** 2020-12-14

**Authors:** Uwe Beekmann, Paul Zahel, Berit Karl, Lisa Schmölz, Friedemann Börner, Jana Gerstmeier, Oliver Werz, Stefan Lorkowski, Cornelia Wiegand, Dagmar Fischer, Dana Kralisch

**Affiliations:** 1 Pharmaceutical Technology and Biopharmacy, Institute of Pharmacy, Friedrich Schiller University, Lessingstraße 8, 07743 Jena, Germany; uwe.beekmann@uni-jena.de (U.B.); paul.zahel@gmail.com (P.Z.); berit.karl@uni-jena.de (B.K.); dagmar.fischer@uni-jena.de (D.F.); 2 Nutritional Biochemistry and Physiology, Institute of Nutritional Sciences, Friedrich Schiller University, Dornburger Straße 25, 07743 Jena, Germany; lisa.schmoelz@uni-jena.de (L.S.); stefan.lorkowski@uni-jena.de (S.L.); 3 Competence Cluster for Nutrition and Cardiovascular Health (nutriCARD) Halle-Jena-Leipzig, Friedrich Schiller University, Dornburger Straße 25, 07743 Jena, Germany; 4 Pharmaceutical and Medicinal Chemistry, Institute of Pharmacy, Friedrich Schiller University, Philosophenweg 14, 07743 Jena, Germany; friedemann.boerner@uni-jena.de (F.B.); jana.gerstmeier@uni-jena.de (J.G.); oliver.werz@uni-jena.de (O.W.); 5 Jena Center for Soft Matter (JCSM), Friedrich Schiller University, Philosophenweg 7, 07743 Jena, Germany; 6 Department of Dermatology, Jena University Hospital, Erfurter Str. 35, 07743 Jena, Germany; c.wiegand@med.uni-jena.de

**Keywords:** bacterial cellulose, wound dressing, anti-inflammatory, drug delivery system, diclofenac, indomethacin, boswellic acid

## Abstract

Natural products suited for prophylaxis and therapy of inflammatory diseases have gained increasing importance. These compounds could be beneficially integrated into bacterial cellulose (BC), which is a natural hydropolymer applicable as a wound dressing and drug delivery system alike. This study presents experimental outcomes for a natural anti-inflammatory product concept of boswellic acids from frankincense formulated in BC. Using esterification respectively (resp.) oxidation and subsequent coupling with phenylalanine and tryptophan, *post*-modification of BC was tested to facilitate lipophilic active pharmaceutical ingredient (API) incorporation. Diclofenac sodium and indomethacin were used as anti-inflammatory model drugs before the findings were transferred to boswellic acids. By acetylation of BC fibers, the loading efficiency for the more lipophilic API indomethacin and the release was increased by up to 65.6% and 25%, respectively, while no significant differences in loading could be found for the API diclofenac sodium. *Post*-modifications could be made while preserving biocompatibility, essential wound dressing properties and anti-inflammatory efficacy. Eventually, in vitro wound closure experiments and evaluations of the effect of secondary dressings completed the study.

## 1. Introduction

Anti-inflammatory strategies for the prophylaxis and the therapy of inflammatory diseases are a highly relevant field of current research. In particular, chronic, age-related, low-grade inflammation increases the risk of serious diseases such as arthritis, cancer, atherosclerosis or chronic wounds [1]. The so-called silent inflammation is related to the inability of the organism to stop and actively resolve inflammatory processes with increasing age. The levels of pro-inflammatory lipid mediators are elevated, and the formation of specialized pro-resolving mediators (SPMs), which are responsible for the inflammatory resolution, is reduced [2]. Anti-inflammatory active pharmaceutical ingredients (APIs), such as ibuprofen [3], diclofenac [4] or indomethacin [5], are well known for treating acute inflammation and reducing the formation of pro-inflammatory prostaglandins, but due to their mode of action they are not able to resolve inflammation [6].

Natural products such as boswellic acids (e.g., 3-O-acetyl-11-keto-boswellic acid, AKBA) from frankincense act at multiple pro-inflammatory targets and have high potential for efficient and safe therapy of inflammation [7]. In comparison to diclofenac or indomethacin, AKBA reduces pro-inflammatory 5-lipoxgenase (5-LO) products [8], cathepsin G [9] and microsomal prostaglandin E_2_ synthase (mPGES)-1 [10]. Recently, we have elucidated the molecular mechanism of AKBA’s anti-inflammatory effects. Thus, for the first time, the crystal structure of 5‑LO with bound inhibitors was imaged [6]. The detailed studies of the enzyme and its allosteric interaction with AKBA clarify that its binding to 5-LO causes a domino effect, which also changes the regio-specificity of the enzyme. In simple terms, this means that AKBA reprograms the inflammatory enzyme into an inflammation-resolving enzyme [6]. This important finding will allow the development of new anti-inflammatory compounds. Unfortunately, AKBA and other boswellic acids are highly lipophilic, with low bioavailability and may induce adverse cytotoxic effects due to the production of metabolites after oral administration [11]. By being applied dermally or transdermally, the bioavailability and safety of these natural substances can be improved. In addition, adverse effects of metabolites of AKBA and other boswellic acids can be reduced by bypassing the first pass effect.

Bacterial cellulose (BC), known as a hydroactive wound dressing, could serve as a drug carrier for dermal or transdermal applications. Bacterial cellulose is a natural hydropolymer produced by different acetic acid bacteria strains (e.g., *Komagateibacter xylinus*, formerly known as *Acetobacter xylinum*) and is suitable as a drug delivery system and wound dressing at the same time [12,13,14,15,16]. This suitability can be explained by its homogenous, open-porous three-dimensional network of cellulose fibers [17], and high liquid absorption and retention capacity [18]. It allows the incorporation and controlled release of APIs, and, at the same time, accomplishes a healing-promoting moist environment. We suggest a natural product concept combining the unique properties of the natural biopolymer BC with the highly effective anti-inflammatory natural substance AKBA. Although the incorporation of lipophilic APIs into BC is still challenging, the hydrophilic characteristic of BC can be overcome by utilizing auxiliary excipients, additives or emulsions [19,20]. Alternatively, modifying the carrier material itself can facilitate the incorporation of lipophilic APIs [21].

The aim of our study was the incorporation of anti-inflammatory lipophilic APIs into *post*-modified BC-based drug delivery systems to investigate improvements of loading capacity and drug release. For this purpose, two approaches of *post*-modifications were carried out and characterized. Esterification has been selected as a promising option to overcome the hydrophilic characteristic of BC [22]. This modification leads to a substitution of hydroxyl groups of cellulose by fewer polar ester groups [23]. Various methods of esterification have been described [24,25,26], but most of those investigations are referred to cellulose nanocrystals (CNCs) and are realized under harsh conditions. Ávila Ramírez and co-authors suggested a simple and mild citric acid-catalyzed surface esterification of CNCs instead [23]. Alternatively, BC can be oxidized with (2,2,6,6-tetramethylpiperidin-1-yl)oxyl (TEMPO) and coupled with lipophilic primary amines or amino acids to reduce its hydrophilic characteristic. Specifically, acetylated BC (AcBC) and TEMPO-oxidized BC (TBC) coupled with lipophilic amino acids such as the more lipophilic phenylalanine (TBCPhe) or the comparably less lipophilic tryptophan (TBCTrp) were chosen for their mild synthesis conditions and biocompatible natures. Loading and release experiments with anti-inflammatory model substances such as diclofenac sodium and indomethacin were carried out at first to gain knowledge necessary for a transfer to natural and highly lipophilic substances such as AKBA. Biocompatibility and effectiveness studies of the prototypes as well as in vitro wound closure experiments were performed. Eventually, application-relevant parameters such as the influence of secondary dressings on the moisture vapor transmission rate (MVTR) and the release were determined and evaluated to establish a natural-based product concept for active BC wound dressings and delivery systems. This may result in novel product development to treat anti-inflammatory diseases.

## 2. Materials and Methods

### 2.1. Preparation of Native and Modified BC

Bacterial cellulose (BC) was produced by static cultivation using the *Komagataeibacter xylinus* strain DSM 14666, deposited at the German Collection of Microorganism and Cell Cultures (DSMZ, Braunschweig, Germany) as described in previous studies [21]. Eventually, samples were sterilized by autoclavation (121 °C, 20 min, 2 bar), and stored (4 °C) or freeze-dried. Freeze-dried BC samples (fd BC) were obtained by lyophilization at −80 °C and 0.01 mbar for 72 h using an Alpha 2–4 LD plus freeze dryer (Martin Christ Gefriertrocknungsanlagen, Osterode am Harz, Germany).

BC esterification was performed according to the method of Ávila Ramírez and co-authors [23]. Therefore, native freeze-dried BC was fully resoaked by acetic anhydride and citric acid (VWR, Radnor, PA, USA) in a glass flask equipped with a reflux condenser. Acetylation was run for two hours at 120 °C. Subsequently, BC samples were purified in several washing steps to remove citric acid and unreacted acetic anhydride, first in acetic acid and then in water for injection (WfI).

BC oxidation was performed by *post*-modification with TEMPO (Alfa Aesar, Haverhill, MA, USA), following Luo and co-authors [27] and Lu and co-authors [28] with a few alterations. TEMPO oxidation of native fd BC was carried out in Na_2_CO_3_/NaHCO_3_ buffer (Carl Roth, Karlsruhe, Germany) at pH 10.5 without controlling the atmosphere. The Na_2_CO_3_/NaHCO_3_ buffer consisted of 0.1 M sodium carbonate solution and 0.1 M sodium hydrogen carbonate solution in a ratio of 4:1. After dissolving sodium bromide (Alfa Aesar, Haverhill, MA, USA) (7.80 g/L) and TEMPO (0.78 g/L), the reaction was started with the addition of 0.81 g/L of sodium hypochlorite solution (13%) (Alfa Aesar, Haverhill, MA, USA). The oxidation was carried out under magnetic stirring (VWR, Radnor, PA, USA) (150 rpm) for 24 h at room temperature at air. The oxidized BC (TBC) was then suspended in 96% ethanol (VWR, Radnor, PA, USA) for 30 min to stop the reaction and subsequently purified in several washing steps in WfI until pH neutrality.

After TEMPO oxidation, TBC coupling reactions with phenylalanine and tryptophan (Alfa Aesar, Haverhill, MA, USA) were investigated and adjusted according to reported procedures [29,30,31]. For this, TBC samples were suspended in 150 mL of 0.85 mmol aqueous phenylalanine or tryptophan solutions, respectively. Then, equimolar amounts of 0.163 g 1-ethyl-3-(3-dimethylaminopropyl)carbodiimide (EDC) (Alfa Aesar, Haverhill, MA, USA) and 0.098 g *N*-hydroxysuccinimide (NHS) (Alfa Aesar, Haverhill, MA, USA) were each dissolved in 10 mL WfI. The NHS solution was slowly pipetted into the reaction mixture under constant stirring. The pH of the solution was adjusted to 5.5–6.0. After 30 min, the reaction was started by the dropwise addition of EDC solution and continued for twelve hours while stirring. The reaction solution was quenched by WfI, and the TBCPhe and TBCTrp samples were purified in several washing steps.

### 2.2. Physical Characterization

Native and *post*-modified BC samples were characterized by various physical parameters, such as diameter, thickness, volume, height, weight, water absorption capacity (WAC), water holding capacity (WHC), solid content (SC), moisture vapor transmission rate (MVTR), compressive strain and weight loss under pressure. All types of characterization were conducted as described [21,32]. Analyses of the morphology of the native and modified BC fiber network were carried out using scanning electron microscopy (SEM) with a Sigma-VP-Scanning Electron Microscope (Carl Zeiss, Oberkochen, Germany), operated at 6 kV using an In-lens detector (Carl Zeiss, Oberkochen, Germany). Freeze-dried BC samples were cut with a razor blade, mounted and sputtered with 5 nm gold (CCU-010 HV Compact Coating System, Safematic, Zizers, Switzerland). For a qualitative hydrophobization assay, native and *post*-modified BC samples were tested by the distribution in test tubes containing two immiscible polar/nonpolar liquid phases (WfI/cyclohexane) (Alfa Aesar, Haverhill, MA, USA). The surface hydrophobicity assay was performed according to previous studies [33]. The level of acetylation was determined via infrared spectroscopy. Fourier transform infrared spectra (FTIR) were taken from freeze-dried BC samples (IR Affinity 1, Shimadzu, Duisburg, Germany) and from *post*-modified BC samples (20 scans for each FTIR spectra). The spectra were analyzed according to Schwanninger et al. [34]. To measure the nitrogen content, BC and modified BC samples were freeze-dried and analyzed by elemental analysis (EURO EA 3000, HEKAtech, Wegberg, Germany) using native BC as blank. To calculate the yield of *post*-modifications, the degree of substitution (DS) was determined (Equation S1). To evaluate the distribution of modified fractions on the surface and in BC fleeces, FTIR imaging was performed. Therefore, freeze-dried native and *post*-modified BC samples were analyzed using FTIR imaging spectroscopy on a Varian 670-IR spectrometer coupled with a Varian 620-IR imaging microscope (Agilent Technologies, Santa Clara, CA, USA). Cross-sections of BC samples were illustrated in intensities at the absorption peaks of interest of respective *post*-modification (native BC and AcBC at 1740 cm^−1^; TBCPhe and TBCTrp at 1605 cm^−1^).

### 2.3. Cell Culture

Cultivation of THP-1 monocytes (ATCC, Manassas, VA, USA) and murine RAW264.7 macrophages were performed as previously described [21]. The human HaCaT keratinocytes were a gift from Prof. Dr. Norbert E. Fusenig (DKFZ, Heidelberg, Germany). HaCaT cells were cultured in Dulbecco’s modified Eagle’s Medium (DMEM, AMIMED, Sigma-Aldrich, St. Louis, MO, USA) supplemented with 1% antibiotic-antimycotic solution (AMIMED, Sigma-Aldrich, St. Louis, MO, USA) and 10% fetal calf serum (FCS, Promocell, Heidelberg, Germany). The cells were cultured for seven days in 75 cm^2^ cell culture flasks (Greiner bio-one, Frickenhausen, Germany) at 37 °C and in humidified atmosphere containing 5% CO_2_ atmosphere.

### 2.4. MTT Assay

In Vitro cytotoxicity was evaluated by incubation of cells with extracts of native and modified samples for 24 h according to previously published procedures [21,35,36]. Absorption of triplicates (of 100 µL each) was measured at 570 nm. The pure cell culture medium was used as negative control. The untreated control was set to 100% and was therefore used as reference.

### 2.5. Scratch Assay

For experiments, HaCaT cells were harvested through trypsin-EDTA (Gibco, Thermo Fisher Scientific, Waltham, MA, USA) treatment, seeded into 6-well culture plates (Greiner bio-one, Frickenhausen, Germany) at a density of 40,000 cells/cm^2^, and cultured for 48 h to confluence. Cell monolayers were scratched with a sterile pipette tip and washed with medium to remove any loose cells. Subsequently, BC samples were placed directly on the scratch and weighed down with a small polypropylene (PP) weight (sterilized Nunc™ Cap Mats, Thermo Fisher Scientific, Waltham, MA, USA). Test specimens were incubated with the cells for 4, 8, 24, and 48 h in complete DMEM (contains 1% antibiotic-antimycotic solution and 10% FCS). Cell scratches cultured in complete DMEM alone served as untreated controls. After the respective incubation periods, BC samples were removed, and cells were stained with hematoxylin and eosin for evaluation. Microscopic evaluation was carried out using the VHX 950F digital microscope (Keyence Deutschland GmbH, Neu-Isenburg, Germany) and images were obtained. Scratch width was determined using the Image J analysis software (1.45 m, NIH, Bethesda, MD, USA). The progression of “healing” of the scratch wound, designated as wound area in [%], was obtained for comparing scratch width at each time point.

### 2.6. Loading and Release

Loading studies with diclofenac sodium and indomethacin (Euro OTC Pharma GmbH, Bönen, Germany) were performed using the absorption loading method as described previously [21,37]. In brief, native and *post*-modified BC samples were deposited independently in 10 mL of 1 mg/mL diclofenac sodium in WfI resp. indomethacin in PEG 400 (Sigma-Aldrich, St. Louis, MO, USA) and maintained for 48 h at room temperature. Samples were shaken at 70 rpm using an orbital shaker (KS 4000 ic control, IKA-Werke, Staufen, Germany). The concentration of the API solution was measured before (B_0h_) and after loading (B_48h_) via ultraviolet–visible (UV/Vis) spectroscopy using the microplate reader Tecan Spark 10M (Tecan Austria, Grödig, Austria) at 274 nm for diclofenac sodium and 321 nm for indomethacin. The amount of the respective API in the respective BC fleece (B_48h_‑B_0h_) was determined from the difference between the two measurements. The comparison of the loading studies was carried out only within the active ingredient group, since effects of the loading medium [37] were to be expected (WfI vs. PEG 400). Instead, the effect of the modifications on the loading and release behavior in relation to native BC was compared. Studies with lipophilic extracts of the gum resin derived from *Boswellia serrata* (*B. serrata* extracts, BSE) were performed according to previous investigations [19].

Release studies under shaking conditions were performed at 70 rpm and 32 °C in 10 mL of phosphate-buffered saline (PBS) pH 7.4 (*n* = 3). After t = 0, 0.25, 0.75, 1, 2, 4, 8, 24, 30, 48, 72 and 120 h, aliquots (0.5 mL) of medium were taken and replaced by fresh and preheated (32 °C) PBS pH 7.4. The released API concentration was measured at each time point via UV/Vis spectroscopy. API losses due to the aliquots taken were considered for determination of relative API amount in relation to the maximally measured API amount per sample after loading (t_0_) of. Subsequently, the time-dependent cumulative API release was determined. All experiments were run in triplicate.

Release studies under vertical diffusion conditions were conducted according to Alkhatib and co-authors [37] in PBS pH 7.4 at 32 °C using a vertical diffusion cell (Franz cell; SES-Analysensysteme, Bechenheim, Germany) with a receiver compartment volume of 12 mL and an effective area for diffusion of 1.77 cm^2^. The receiver compartment was magnetically stirred and tempered to 32 °C by a water jacket. Loaded (modified) BC was placed in the donor compartment on a specifically designed aluminum foil cross instead of a membrane in order to allow maximum contact area between the (modified) BC sample and the release medium (one-sided). The samples were placed in a way that they had direct contact with the release medium. After the same time points as the ones chosen for the release studies under shaking conditions, aliquots (0.5 mL) of the receiver compartment were withdrawn and replaced by fresh and preheated (32 °C) PBS pH 7.4. The amount of released API was quantified using the UV/Vis spectrophotometric measurement as described above.

Release studies with various secondary dressings were also performed using Franz cells. Native BC samples were loaded with 1 mg/mL indomethacin solution as described above and placed onto the Franz cells. Contrary to fixation with the donor compartment, BC samples were fixed with secondary dressings (Figure S1). Paraffin gauze Jelonet^®^, occlusive dressing Opsite^®^ Flexifix and gauze bandage Ypsiflex^®^ were used. The further procedure of the experiment was analogous to previous Franz cell experiments [19].

### 2.7. Proof of Efficacy

#### 2.7.1. Isolation of Human Polymorphonuclear Leukocytes (PMNLs) and Human Platelets

Leukocyte concentrates were prepared from peripheral blood obtained from healthy human adult donors that had received no anti-inflammatory treatment for the previous 10 days (Institute of Transfusion Medicine, University Hospital Jena, Germany). The approval for the protocol was given by the ethical committee of the University Hospital Jena and all methods were performed in accordance with the relevant guidelines and regulations. To isolate polymorphonuclear leukocytes (PMNLs) and platelets, the leukocyte concentrates were mixed with dextran (dextran from leuconostoc spp. MW ~40,000 g mol-1, (Sigma-Aldrich, St. Louis, MO, USA) for sedimentation of erythrocytes; the supernatant was centrifuged on lymphocyte separation medium (Histopaque^®^-1077, Sigma-Aldrich, St. Louis, MO, USA). For isolation of platelets, the supernatants of the Nycoprep cushion centrifugation were mixed with PBS pH 5.9 (3:2 *v*/*v*), centrifuged (2100× *g*, 15 min, 20 °C), and the pelleted platelets were resuspended in PBS pH 5.9/0.9% NaCl (1:1, *v*/*v*). Washed platelets were finally resuspended in PBS pH 7.4 and 1 mM CaCl_2_. For isolation of PMNLs, the contaminating erythrocytes in the cell pellet of the Nycoprep cushion centrifugation were removed by hypotonic lysis using water. PMNLs were then washed twice in ice-cold PBS pH 7.4 and finally resuspended in PBS.

#### 2.7.2. Activity Assay of COX-1 in Human Isolated Platelets

To ensure the biological effectiveness of the active substances after release from BC and *post*-modified BC, COX-1 activity assays in human platelets were carried out according to Pein and co-authors [38] as described in previous studies [21].

#### 2.7.3. Activity Assay of 5-LO in Human PMNLs

For evaluation of the effects on 5-LO product formation in human PMNLs, cells (5 × 10^6^ cells/mL) were preincubated with test compounds or media for 10 min at 37 °C. Cells were then stimulated with 2.5 µM Ca^2+^-ionophore A23187 (Cayman Chemical, Ann Arbor, MI, USA) plus 2 µM arachidonic acid for 10 min, and then the incubation was stopped with 1 mL ice-cold methanol containing 200 ng PGB_1_ as internal standard. Samples were subjected to solid phase extraction and formed 5-LO products, and were separated and analyzed by reversed phase high-performance liquid chromatography (RP-HPLC) as described [39].

#### 2.7.4. Griess Assay (Nitric Oxide Measurement)

Murine RAW264.7 macrophages were treated with extracts of *post*-modified and native BC samples as described earlier [21,40]. Aliquots of incubation medium were mixed with water and Griess reagent and measured at 544 nm with a FLUOstar omega microplate reader (BMG Labtech, Ortenberg, Germany) after an incubation in the dark for 30 min. MARS data analysis software version 2.41 was used for analyses. A standard dilution row of NaNO_2_ was used as reference.

## 3. Results and Discussion

### 3.1. Modification of BC

**Figure 1:**
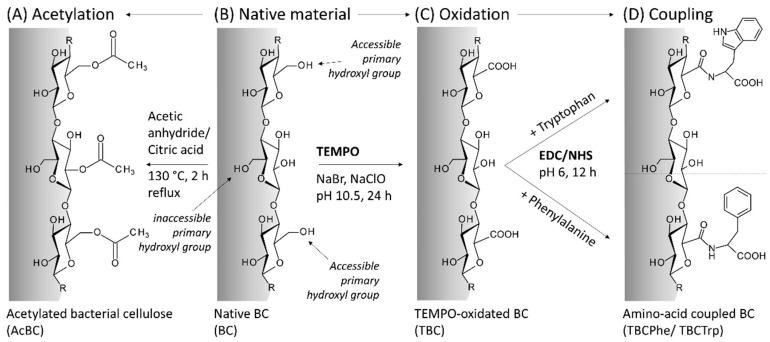
Overview of investigated bacterial cellulose (BC) *post*-modifications starting from native BC (**B**). (**A**) Acetylation of native BC; (**C**) TEMPO-oxidation of native BC; (**D**) Coupling of TEMPO-oxidized BC with Tryptophan or Phenylalanine, respectively.

Developing BC-based drug delivery systems is still challenging due to the generally lipophilic properties of many APIs. Modifying BC by chemical modification can tailor this biomaterial to an application-specific purpose and facilitate lipophilic API incorporation. Two approaches of BC *post*-modifications resulting in three different types of modified BC were carried out and characterized (Figure 1).

By using the free radical TEMPO and sodium bromide, the oxidation of the hydroxyl functions of native BC by sodium hypochlorite can be modified at pH 10 and room temperature via aldehydic and ketonic intermediates to carboxyl functions [41]. The simplicity, efficiency and mild reaction conditions of the process qualifies the TEMPO oxidation as basis for further conjugations of various compounds to BC [29]. For TBC derivatization, the conjugation of the created carboxyl functions with lipophilic primary amines such as amino acids is one of the various options. In this study, the proteinogenic amino acids phenylalanine and tryptophan were chosen. They are, according to their physiological occurrence, biocompatible, readily available and have a more lipophilic characteristic than cellulose due to their aromatic side chains. Furthermore, the esterification reaction is also known for its mild reaction condition and single-step characteristic. The reaction realizes the introduction of acetyl function into the network by a simple direct solvent-free route catalyzed by citric acid. [42].

**Figure 2:**
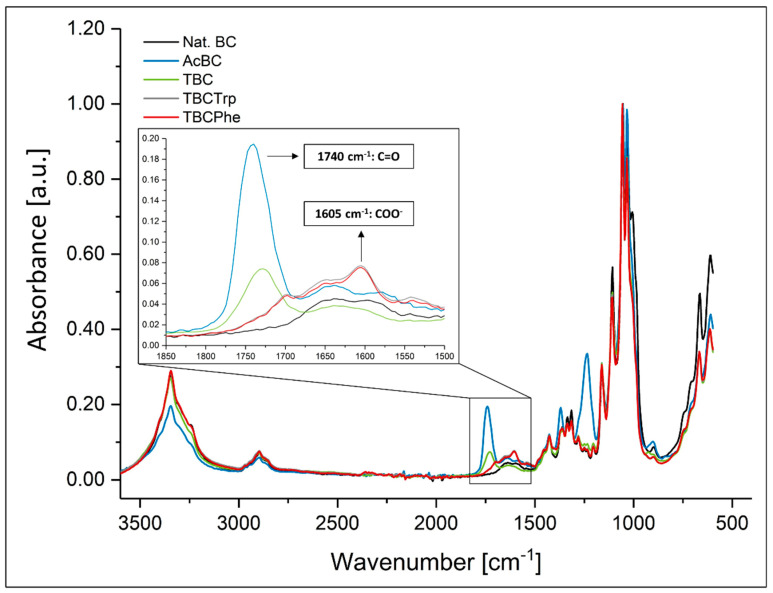
Infrared spectra of native BC and *post*-modified BC samples acetylated BC (AcBC), TEMPO-oxidized BC (TBC), lipophilic phenylalanine (TBCPhe) and lipophilic tryptophan (TBCTrp) (*n* = 3).

After the preparation of native and *post*-modified BC, the samples were characterized. Figure 2 depicts Fourier transform infrared spectroscopy (FTIR) spectra of *post*-modified BC samples (acetylated BC (AcBC), TEMPO-oxidized BC (TBC), TEMPO-oxidized BC coupled with phenylalanine (TBCPhe), TEMPO-oxidized BC coupled with tryptophan (TBCTrp)) compared to native BC (nat. BC).

FTIR spectra of AcBC revealed significant differences to spectra of native BC which confirmed that acetyl functional groups were introduced into BC fleeces. Esterification could be confirmed by the C=O and C-O stretching observed at 1740 and 1234 cm^−1^, respectively, and C-H bending at 1369 cm^−1^. Additionally, the decrease in the absorption peak at 3345 cm^−1^ is referred to successful esterification. These results were in line with earlier investigations—e.g., in the context of CNC modification [22].

Whereas native BC weakly absorbs in the range of 1500 to 1900 cm^−1^ at 1650 cm^−1^ due to hydroxyl diffraction vibrations of the remaining water, TBC shows characteristic absorption peaks of carbonyl stretching vibrations at 1600 cm^−1^ for the sodium carboxylate function and 1735 cm^−1^ for the free carboxylic acid [43]. Both absorption peaks are thus in equilibrium with each other and are signals of the same functional group. Their intensity depends on the *post*-treatment of oxidized cellulose (protonation or deprotonation of free carboxyl groups). The carboxyl groups introduced into BC by TEMPO oxidation formed the basis for functionalization with lipophilic compounds containing primary amines. Carboxylic acids are activated by 1-ethyl-3-(3-dimethylaminopropyl)carbodiimide (EDC) to form an unstable O-acylisourea. This intermediate reacts to nucleophilic attack by primary amines to form an amide bond. *N*-Hydroxysuccinimide was added to improve the yield by the formation of NHS esters with EDC, which are more stable to hydrolysis than the intermediates formed exclusively from EDC and carboxyl groups. By this reaction, the carboxyl functions of TBC can be functionalized by coupling with an EDC/NHS system via an amide bond with the lipophilic amino acids phenylalanine or tryptophan. The FTIR spectra depict a clear difference between TBC, TBCPhe, TBCTrp and native BC (Figure 2). The carbonyl absorption peak (1735 cm^−1^) of TBC disappeared in the FTIR spectra of TBCPhe and TBCTrp due to the formation of amide bonds. However, the FTIR spectra of TBCPhe show a broader absorption peak in the range of 1540 to 1680 cm^‑1^, which can be assigned to an asymmetric carbonyl valence vibration of the carboxylate function of the coupled phenylalanine [29,44,45]. The signals observed coincide with previously published studies and indicate successful functionalization. Huang et al. also described the coupling of TBC with phenylalanine and tryptophan observing intense carbonyl absorption peaks of the coupled cellulose in the range of 1540 to 1680 cm^−1^ [29]. TBCTrp samples show comparable signals to TBCPhe in FTIR spectra. However, a broader “shoulder” of this absorption peak is noticeable at 1605 cm^−1^, which can be assigned to the formation of hydrogen bonds between free hydroxyl functions and carboxylate groups of the coupled amino acids [45]. The degree of substitution (DS) for TBCPhe and TBCTrp was calculated using Equation S1 based on results of elemental analyses. Based on the amount of nitrogen measured, a DS of 0.0002 ± 0.0001 for native BC as negative control, 0.044 ± 0.004 for TBCPhe and 0.043 ± 0.008 for TBCTrp was determined. The DS of AcBC could not be calculated by this method due to inaccuracies in the method (data not shown).

**Figure 3:**
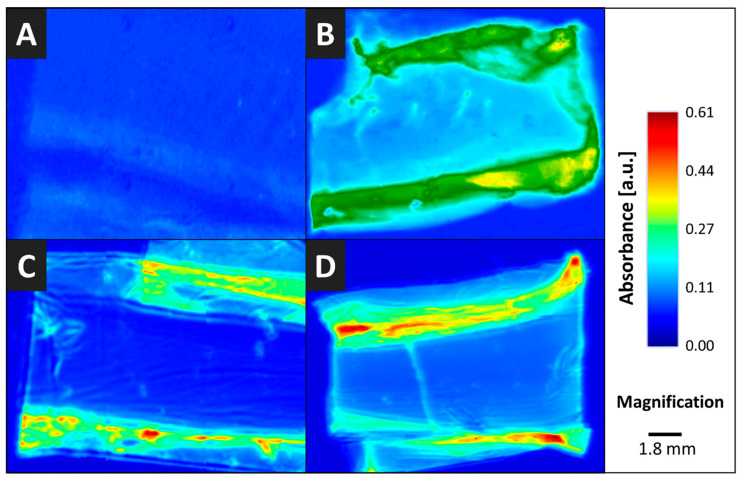
IR imaging pictures of native and *post*-modified BC cross-sections for (**A**): native BC and (**B**): AcBC at 1740 cm^−1^, (**C**): TBCPhe and (**D**): TBCTrp at 1605 cm^−1^.

To clarify the distribution of the modification in BC pellicles, FTIR imaging was performed with native and *post*-modified BC cross-sections (Figure 3). FTIR imaging spectrometers combine the spectral with lateral information, since spectra are acquired in parallel and images are collected for each spectral data point [46]. Coupling IR imaging spectrometers with microscopes gives diffraction-limited resolution in the five- to ten-micrometer range. The analysis in Figure 3 confirmed that the *post*-modifications investigated cause mainly surface modifications. Higher absorption peaks at respective identification peaks in the peripheral area were found for each modification investigated. This observation may be explained by the higher accessibility of the outer parts of the BC network for reactants as an effect of time-dependent diffusional processes. The modification of the surface is not uniform, but the trend of the surface being significantly more modified than the inner side is clearly visible. Areas with a higher extent of modification (and related absorbance) are hypothesized to allow the incorporation of a higher amount of lipophilic active ingredients.

Water absorption capacity, WHC and SC were investigated for physical characterization. The parameters of *post*-modified BC must remain comparable to native BC because its high water content is known to be beneficial for wound treatment. Figure S2 depicts the properties of native BC and *post*-modified BC. WHC was slightly affected by the different *post*-modifications (range: 669% to 861%). In the case of WAC and the SC, the acetylation reaction caused a higher deviation than oxidation and subsequent amino acid coupling. AcBC showed decreased WAC (5562 ± 549% WAC, 1.82 ± 0.18% SC) in comparison to native BC (6839 ± 733% WAC, 1.48 ± 0.16% SC), whereas TBCPhe (6834 ± 825% WAC, 1.49 ± 0.18% SC) and TBCTrp (6655 ± 751% WAC, 1.52 ± 0.17% SC) have comparable WAC and SC characteristics compared to each other and to native BC.

**Figure 4:**
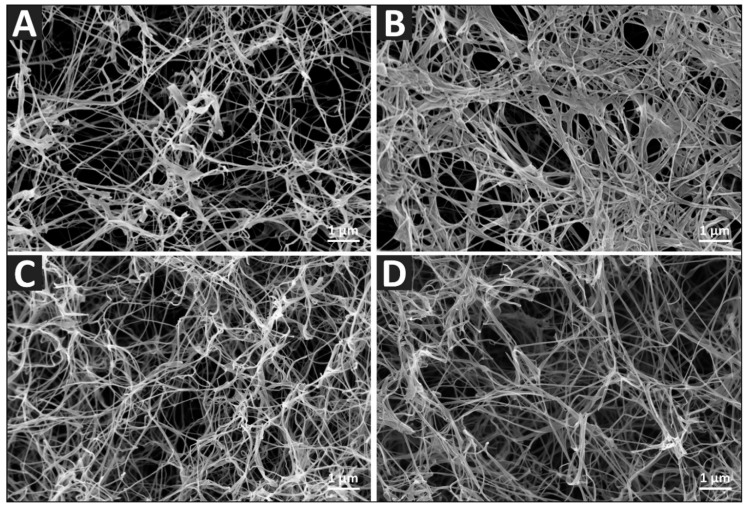
BC network morphology depicted by scanning electron microscopy (SEM). Cross-sections of freeze-dried samples of (**A**): native BC, (**B**): AcBC, (**C**): TBCPhe, (**D**): TBCTrp were sputter-coated with gold before imaging.

The evaluation of the mechanical characteristics of native and *post*-modified BC (Figure S3) revealed comparable trends. AcBC (76.36 ± 0.35%) differs significantly (*p* = 0.008) from native BC (61.38 ± 2.09%) regarding the compressive strain, whereas TBCPhe (65.16 ± 4.12, *p* = 0.332) and TBCTrp (69.69 ± 4.06, *p* = 0.083) show commensurable characteristics. AcBC samples showed an increased compressive strain due to the different network structure which could be shown by SEM images (Figure 4). The SEM images revealed partial collapse of the acetylated cellulose fiber network (Figure 4B). A stronger cohesion of the fibers was observed compared to native BC (Figure 4A), TBCPhe (Figure 4C) and TBCTrp (Figure 4D). Due to the decreased hydrophilic character of cellulose fibers in the AcBC network, smaller amounts of water were suggested to be incorporated, strengthening the interaction of the fibers and leading to cohesion. Qualitative experiments further confirmed the decrease in the hydrophilic characteristic of the *post*-modified samples. For example, it could be shown that the distributions in test tubes containing two immiscible polar/nonpolar liquid phases (WfI/cyclohexane) of native BC and AcBC are different (Figure S4). Whereas native BC sank into the aqueous phase, AcBC remained in the interphase of lipophilic (cyclohexane) and hydrophilic (WfI) phase, which means that the network has become more hydrophobic. Comparable results could be found for TBCPhe and TBCTrp.

The MVTR is another important application-relevant parameter to be characterized for the development of wound dressings. For an ideal gas and liquid exchange through the wound dressing, the range of the MVTR should be between 2500 and 3000 g·m^−2^·24 h^−1^ [47,48]. However, not only the MVTR of the primary wound dressing is decisive, a secondary wound dressing, likely applied in the case of BC-based dressings, can also influence the MVTR or change the MVTR of the primary wound dressing. For this reason, the MVTR of the *post*-modifications compared to the native BC was examined, and the influence of various secondary dressings on the MVTR was evaluated using the example of the native BC. It is particularly important to consider and assess the MVTR when the material is functionalized, because changing properties of cellulose fibers and the network may have an influence on water vapor permeability. In Figure S5, the MVTR of native BC compared to *post*-modifications (Figure S5A) and the impact of secondary dressings on the MVTR of native BC (Figure S5B) are represented. Interestingly, the MVTR of *post*-modified BC is quite comparable to each other and to native BC (3262 ± 166 g·m^−2^·24 h^−1^ for native BC, 3320 ± 64 g·m^−2^·24 h^−1^ for AcBC, 3089 ± 167 g·m^−2^·24 h^−1^ for TBCPhe). TBCTrp (2702 ± 188 g·m^‑2^·24 h^‑1^) merely showed a decrease in MVTR. Despite hydrophobization, which affects the loading capacity and release of lipophilic APIs, MVTR was only slightly influenced. However, secondary dressings can change the conditions. To evaluate the influence of secondary dressings on the MVTR, different types were used. A classical gauze bandage (Ypsiflex^®^), an occlusive dressing (Opsite^®^ Flexifix) and a paraffin gauze (Jelonet^®^) were applied exemplarily in combination with native BC (Figure S5B). Although the gauze bandage (Ypsiflex^®^) affected the MVTR (2850 ± 147 g·m^−2^·24 h^−1^) the least compared to native BC, it nevertheless was found unsuitable as a cover of drug delivering BC systems because of its soaking properties. The MVTR of BC plus occlusive dressing (Opsite^®^ Flexifix, 990 ± 54 g·m^−2^·24 h^−1^) and BC plus paraffin gauze (Jelonet^®^, 1403 ± 190 g·m^−2^·24 h^−1^) was greatly reduced by the secondary dressing. Ultimately, the use of secondary dressings is necessary to fix the primary dressing, which means that the product development should ensure that basic material properties such as the MVTR are only slightly influenced.

### 3.2. Biocompatibility

Macrophages were used to test biocompatibility of extracts of BC and *post*-modified BC, as they are important in the context of inflammation and wound healing and are more sensitive to MTT (3-(4,5-dimethylthiazol-2-yl)-2,5-diphenyltetrazolium bromide) viability assays than fibroblasts (3T3) [49], or epithelial cancer cells [50]. The extracts of BC samples were prepared according to previous studies [21,35,36]. RAW264.7 (murine macrophage-like cells) and THP-1 (human monocytic/macrophage-like cells) were used. No cytotoxic effects were observed for the tested extracts (Figure S6). Furthermore, the viability of cells treated with modified BC extracts was akin to that of cells cultured with native BC extracts, suggesting that selected *post*-modifications of BC did not alter the biocompatibility of the material. Eventually, these results can be compared with previous investigations [36] and results from other groups [51], which also evaluated, e.g., the cytotoxicity of TEMPO-oxidized cellulose nanofibers.

### 3.3. Scratch Assay

**Figure 5:**
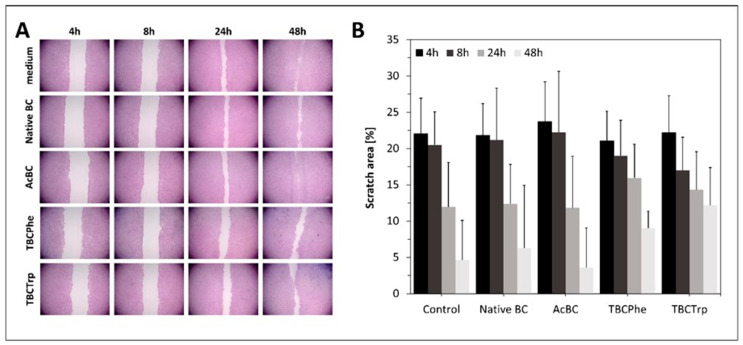
(**A**): Images of measurements of cell migration in in vitro scratch assay with HaCat cells after 4, 8, 24 and 48 h. Cell culture medium was used as control. The rate of migration was measured by quantifying the total distance that from the edge towards the center of the scratch. (**B**): Percental scratch area of native and *post*-modified BC in in vitro wound closure experiments (mean value (MV) ± standard deviation (SD); *n* = 6; m = 2).

To compare the influence of native and *post*-modified BC on wound healing, scratch assays with human keratinocytes were performed in vitro. Mechanical scratch wounding of cell monolayers can be used to study cell migration and layer regeneration by cell proliferation [52,53,54]. Previously, it has been shown that samples of nonadherent dressings can be directly applied to fibroblast monolayers for evaluation of effects on cell behavior [55]. Hence, BC samples were used directly on the cell scratch in this study. In Vitro wound closure was investigated at 4, 8, 24, and 48 h post injury time (Figure 5). Native BC, AcBC and TBCPhe had no distinct effect on the scratch closure compared to the medium control in vitro, but AcBC increased the scratch closure over 48 h compared to native BC and the other *post*-modifications, which constitutes another positive factor choosing acetylation *post*-modification. Compared to this, TBCTrp significantly (*p* = 0.008) decreased the scratch closure over 48 h compared to the medium control (Figure 5B). This outcome suggests that the BC modification TBCPhe may have a negative effect on wound healing.

### 3.4. Loading and Release Studies

**Figure 6:**
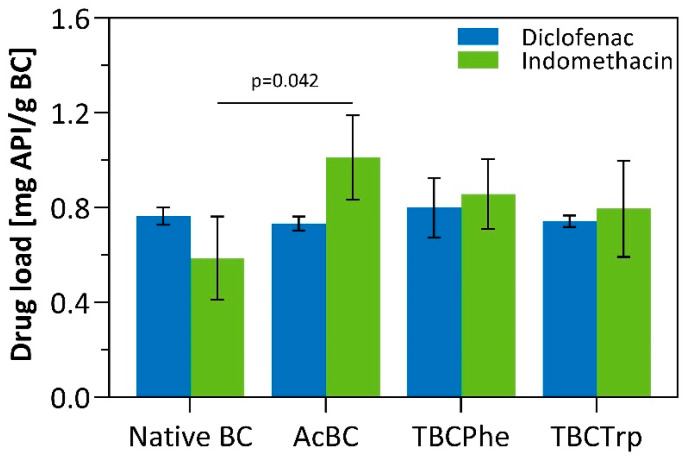
Comparison of drug load (mg active pharmaceutical ingredient (API)/g BC) of diclofenac sodium and indomethacin for native BC and *post*-modified BC (MV ± SD; *n* = 3). A significant improvement of indomethacin incorporation compared to native BC was found for AcBC (*p* = 0.042).

The loading and release behavior of native and *post*-modified BC was investigated using two lipophilic model drugs—diclofenac sodium (log P = 4.06, solubility in water = 0.0048 mg/mL, solubility in PBS pH 7.4 = 9 mg/mL) and indomethacin (log P = 4.27, solubility in water = 0.0024 mg/mL, solubility in PBS pH 7.4 = 3.17 mg/mL). In the literature, many studies on BC-based drug delivery systems can be found [15,16]. Most of these studies address the short-term release of hydrophilic small molecules [56,57,58,59,60,61], whereas only a few described long-term release [37,62] or the incorporation of large molecules [32,63,64,65]. In most of these studies, the release profile of BC can be characterized as biphasic with an initial burst and a plateau phase. In this study, the effect of a hydrophobized cellulose network on lipophilic API incorporation and drug release was investigated with nonsteroidal anti-inflammatory model drugs (NSAIDs) to provide the experimental basis for the incorporation of boswellic acids. To incorporate diclofenac sodium and indomethacin by absorption loading, BC and *post*-modified BC were incubated under gentle shaking to support homogenous uptake. The results on the uptake of diclofenac and indomethacin are depicted in Figure 6. On the one hand, differences between both APIs, characterized by different log *P* values and water solubility, and, on the other hand, effects of *post*-modified BC compared to native BC, were evaluated. Whereas *post*-modifications of BC had no significant impact on the uptake of diclofenac sodium, indomethacin load was increased up to 1.01 ± 0.18 mg API/g BC (65.6%) for AcBC, 0.86 ± 0.15 mg API/g BC (59.4%) for TBCPhe and 0.80 ± 0.20 mg API/g BC (59.0%) for TBCTrp compared to 0.59 ± 0.18 mg API/g BC for native BC. Thus, AcBC, TBCPhe and TBCTrp facilitate the incorporation of the more lipophilic, less water-soluble API indomethacin due to stronger van der Waals forces and hydrophobic interactions resulting from the *post*-modifications. In contrast, the same increase in uptake could not be observed for the more hydrophilic and water-soluble API diclofenac sodium. The partial hydrophobization of the BC fiber network does not seem to affect the strong hydrogen bonds between cellulose and the carboxy groups of diclofenac sodium.

**Figure 7:**
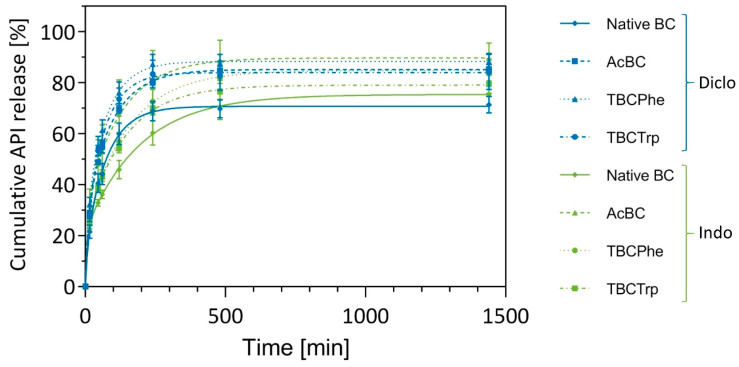
Cumulative release profiles of diclofenac sodium and indomethacin loaded native and *post*-modified BC at 32 °C. Samples were taken at specified time points, and the amount of API was quantified (MV ± SD; *n* = 3).

Continuing with the loaded modified BC-based drug delivery systems (Figure 6), release experiments under shaking conditions were run to assess differences between both APIs and BC samples analogous to loading experiments (Figure 7). A faster release for both APIs from modified BC samples compared to native BC was found. Furthermore, all *post*-modified BC samples increased the amount of released API (e.g., from 71.4 ± 3.2% for native BC to 88.3 ± 3.2% for TBCPhe of diclofenac or from 75.4 ± 4.3% for native BC to 89.6 ± 6.1% for AcBC of indomethacin). This observation applies to both active substances and may be explained by the higher degree of functionalization on the carrier material surface and the likeliness that the lipophilic drug was mostly incorporated in this area.

To transfer these findings to boswellic acid incorporation, loading experiments with the most promising *post*-modification (esterification) compared to native BC and lipophilic extracts of the gum resin derived from *Boswellia serrata* (“Frankincense”, *B. serrata* extracts, BSE) were carried out. Initial findings showed that AcBC, as in the model studies, can improve the loading efficiency of lipophilic compounds such as KBA (log P = 7.10) and AKBA (log P = 8.44) in a similar way as nanoemulsions [19]). However, the first Franz cell release experiments with native BC and AcBC samples loaded with these lead compounds have revealed a lower cumulative release of ABKA and KBA (data not shown) compared to a formulation in nanoemulsions [19]. Further studies are therefore needed to investigate the mechanisms behind release vs. retaining of highly lipophilic drugs within the (modified) BC carrier material prior further optimization of *post*-modification strategies.

**Figure 8:**
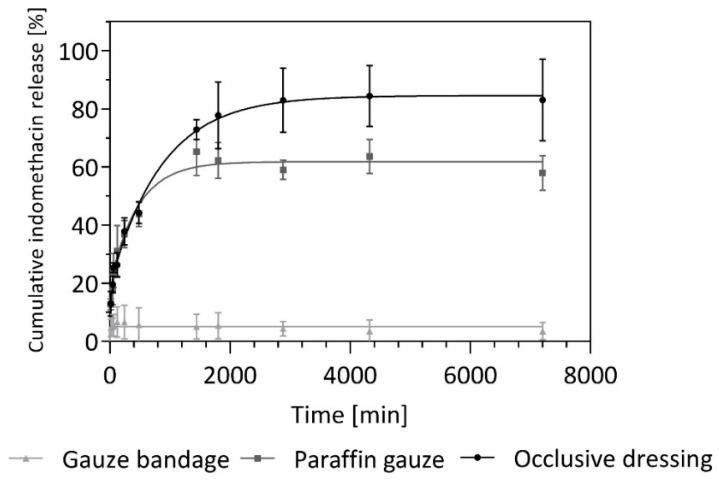
Exemplary representation of API release (here: indomethacin) with consideration when using different secondary dressings (gauze bandage, paraffin gauze, occlusive dressing) on top of BC wound dressing in Franz cell experiments (MV ± SD; *n* = 3).

The clinical use of BC-based drug delivery systems and wound dressings made of BC is accompanied in practice by various secondary dressings for fixation in accordance with the recommendations of different manufacturers. Hence, in in vitro Franz diffusion cell release experiments, three different secondary dressings were tested on their influence on API release from native BC. Exemplarily, indomethacin was chosen as lipophilic model API. Figure 8 shows the release profiles obtained for gauze bandage (Ypsiflex^®^), paraffin gauze (Jelonet^®^) and occlusive dressing (Opsite^®^ Flexifix) as the percental cumulative indomethacin release over time and reveals clear differences between the release profiles, depending on the respective secondary dressings. The release profile of BC plus Opsite^®^ Flexifix is most comparable to the profile of native BC without a secondary dressing. As described above, a large amount of the API was released in a rapid burst phase within the first 24 h, followed by a plateau phase (biphasic release system). In comparison, the highest amount of API was released to the BC-released medium covered with an occlusive dressing. As a second dressing, fixation with paraffin gauze was examined according to the application instructions [66,67] of various BC-based wound dressings. This lattice-like tissue impregnated with paraffin (petroleum jelly) does not form a continuous occlusive layer but allows the drug delivery system to have partial direct air contact with the environment. In the Franz cell diffusion experiments, there was no difference in the BC covered with occlusive dressing during the initial phase of up to 480 min. From a duration of 24 h onwards, however, there was first a decrease in the release rate followed by the final plateau setting. About 60% of the loaded API was released on average from BC plus paraffin gauze. A classic gauze dressing (Ypsiflex^®^) was examined as the third secondary dressing. This should serve as a reference and is currently not recommended for fixation for commercial BC products. A significantly reduced release can be observed when covered with gauze bandage. After a short initial phase of 120 min, the release almost stopped. Only about 7% of the loaded amount of API was released. One explanation for this could be a pronounced capillary effect of the cotton fabric, which causes the loading solution to be soaked into the secondary dressing and thus lose contact with the release medium.

Taking the results of MVTR and drug release investigations together, using a secondary dressing with low soaking properties but high MVTR seems to be favorable. Among those investigated, paraffin gauze was found to be a better option than gauze or occlusive bandages, but further investigations are needed to find an optimal secondary dressing for BC-based drug delivery systems.

### 3.5. Proof of Efficacy

To demonstrate the efficacy of the drug delivery system, we performed a COX-1 activity assay using human isolated platelets for investigation of the COX-inhibitory action of released diclofenac sodium and indomethacin. Native BC and modified BC samples were loaded with the respective API, which was afterwards released into the respective release media. We then studied the media, potentially containing the APIs for their biological effectiveness in terms of COX-1 inhibition in the platelets. As can be seen from Figure S7, all media efficiently inhibited the activity of COX-1, suggesting that diclofenac and indomethacin were released and accomplished COX-1 inhibition.

**Figure 9:**
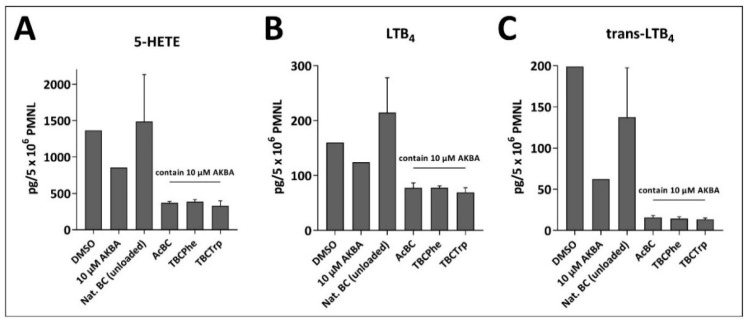
Effects of media derived from 3-O-acetyl-11-keto-boswellic acid (AKBA)-containing BC samples on 5-lipoxgenase (5-LO) activity in human polymorphonuclear leukocytes (PMNLs). Freshly isolated PMNLs were preincubated with the compounds or media for 10 min at 37 °C and then activated with 2.5 µM A23187 plus 2 µM arachidonic acid. After 10 min at 37 °C, the formed 5-LO products 5-HETE (**A**), LTB_4_ (**B**) and *trans*-isomers of LTB_4_ (**C**) were determined by reversed phase high-performance liquid chromatography (RP-HPLC). Data are given as MV ± SD, *n* = 3.

To evaluate the transferability of this successful proof of efficacy to natural products, further experiments were performed in human PMNLs (Figure 9). It could be shown that the release of AKBA from modified BC into the release medium conferred the media potent inhibition of 5-LO activity in PMNLs. Thus, the media derived from AcBC, TBCPhe and TBCTrp containing 10 µM AKBA, all suppressed the formation of the 5-LO-derived products 5-HETE, LTB_4_ and *trans*-LTB_4_ isomers in PMNLs. In contrast, the medium obtained after incubation of unloaded native BC was inactive, as expected.

**Figure 10:**
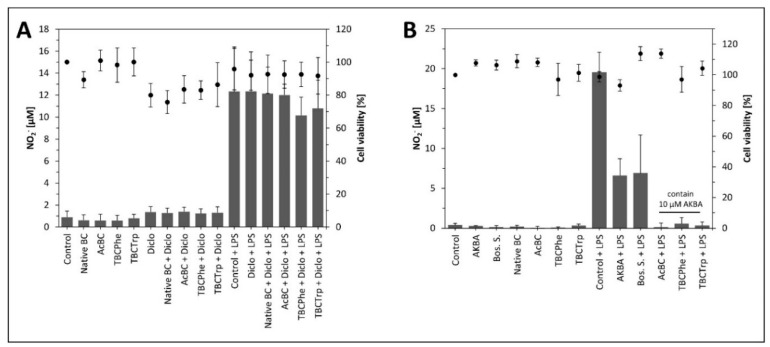
Griess assay using RAW264.7 cells testing BC and modified BC samples to measure nitrite in cell culture supernatant. Cells were incubated with 50% BC/Dulbecco’s modified Eagle’s Medium (DMEM) extracts for four hours followed by a second incubation with the respective extracts and lipopolysaccharide (LPS) for the indicated samples ((**A**) unloaded BC and BC loaded with diclofenac sodium, (**B**) unloaded BC and BC loaded with AKBA or frankincense extract). Cell viability was tested using the MTT (3-(4,5-dimethylthiazol-2-yl)-2,5-diphenyltetrazolium bromide) assay as described above. Data are shown as MV ± SD of four biological replicates.

The native and *post*-modified BC dressing materials were tested regarding their inflammatory effects using a Griess assay [68]. The formation of nitric oxide was measured in vitro using murine macrophages. Modified, unloaded BC and BC loaded with diclofenac sodium, did not cause an activation of murine RAW264.7 macrophages, as no nitric oxide was released. At the same time, the BC and BC loaded with diclofenac did not block the lipopolysaccharide (LPS)-induced nitric oxide formation in murine RAW264.7 macrophages as expected (Figure 10A). In contrast, extracts of BC and modified BC samples, which have been previously loaded with 10 µM AKBA (Figure 10B) showed a remarkable effect. The blocking of LPS-induced nitric oxide release by these BC extracts exceeded the potential of AKBA or frankincense extract to block the LPS-induced nitric oxide burst. Thus, the combination of substance (AKBA or frankincense extract) and carrier (BC or modified BC) enhances the potential of the substance.

## 4. Conclusions and Outlook

In this study, we have shown that *post*-modifications of BC can help to facilitate lipophilic API incorporation (e.g., indomethacin) with tailor-made changes in material properties for BC-based medical and/or pharmaceutical applications. The esterification of BC seems to be the most promising *post*-modification because of its single-step reaction characteristic, mild synthesis conditions, natural origin of reaction components and nontoxicity of the catalyst. We have also shown for the first time that selected *post*-modifications of planar BC wound dressings are feasible, can be characterized mainly as surface modifications and can preserve desired material properties for wound management, such as the MVTR, WAC and biocompatibility. In Vitro wound closure experiments pointed out that selected BC *post*-modifications do not affect scratch closure of keratinocytes, except for TBCTrp. Loading and release studies under shaking conditions with lipophilic model APIs, diclofenac and indomethacin, revealed a faster release and an increased amount of released API for BC *post*-modifications. A high efficacy of diclofenac and indomethacin regarding inhibition of COX-1 in platelets after release was found in in vitro experiments for both, native and *post*-modified BC samples. Next, AKBA was found to be highly effective in reducing inflammation mediators and LPS-induced nitric oxide generation after its release from native and *post*-modified BC examined in human PMNLs and by Griess assays, respectively. Although further studies are needed to optimize the cumulative release, the potential of modified BC as a carrier for highly potent natural anti-inflammatory substances such as AKBA seems to be quite high. The results presented here may pave the way for the development of all-in-one natural, active wound dressings and transdermal patches suited to treat and resolve inflammation.

However, for further design studies, the considerable effect of secondary dressings on the resulting release rates should be considered from the beginning. Based on this, uniform recommendations for the fixation of the drug carrier need to be found to ensure therapy success in the future. The present results indicate that further studies should focus on paraffin gauzes or even more occlusive dressings, as long as a sufficient MVTR can be maintained.

## Supplementary Materials

The following are available online at https://www.mdpi.com/2079-4991/10/12/2508/s1: Figure S1: Fixation of indomethacin-loaded native BC samples with secondary dressings: (A) without secondary dressing, (B) paraffin gauze Jelonet^®^, (C) occlusive dressing Opsite^®^ Flexifix, and (D) gauze bandage Ypsiflex^®^ onto Franz cells. Release experiments with prepared cells were run in triplicate.; Figure S2: Water absorption capacity (WAC), water holding capacity (WHC) and solid content (SC) of native BC and post-modified BC samples (AcBC, TBCPhe, TBCTrp) (mean ± standard deviation; *n* = 8).; Figure S3: Reduction in weight and compressive strain (ε) of native BC compared to post-modified BC. Samples were burdened in perpendicular direction for ten minutes at 32 °C using a test weight of 400 g. Results are expressed as mean of three BC fleeces ± standard deviation.; Figure S4: Qualitative hydrophobicity assay exemplarily depicted for native (A, B, C) and modified BC (A: Acetylated BC (AcBC), B: TEMPO-oxidized BC coupled with phenylalanine (TBCPhe), C: TEMPO-oxidized BC coupled with tryptophan (TBCTrp)) samples. The distribution of the samples in test tubes containing two immiscible polar/nonpolar liquid phases (water/cyclohexane) was evaluated according to previous studies (Ávila Ramírez et al., 2014). The water phase is on the bottom side of the cyclohexane phase; Figure S5: A: Moisture vapor transmission rate (MVTR) of BC and post-modified BC samples. B: MVTR of native BC combined with different secondary dressing. Data are given as mean ± standard deviation (*n* = 5 for each group); Figure S6: Cytotoxic effects of BC and modified BC samples on RAW264.7 cells and THP-1 cells after 24 h incubation time using 50% BC/RPMI-extracts for THP-1 cells and 50% BC/DMEM for RAW264.7 cells. The respective cell culture medium without BC extracts was used as control. Cell viability [%] was determined by MTT (3-(4,5-dimethylthiazol-2-yl)-2,5-diphenyltetrazolium bromide) assay and is shown as mean ± standard deviation of three biological replicates.; Figure S7: Inhibition of COX-1 activity (expressed as 12-hydroxyheptadecatrienoic acid (12-HHT) formation) in human platelets by released diclofenac sodium and indomethacin. Isolated platelets were incubated with the media obtained from incubation of the various BCs for five minutes. Then, 5 µM of arachidonic acid were added, and after five minutes at 37 °C, the formation of 12 HHT was determined. Data given as mean ± standard deviation; *n* = 3 for each group; Equation (S1): Equation for yield calculations of TBCPhe and TBCTrp (DS_Phe_ = Degree of substitution phenylalanine, DS_Trp_ = Degree of substitution tryptophan, M_AGU_ = Molar mass anhydroglucose unit, w_N_ = Mass fraction nitrogen, M_N_ = Molar mass nitrogen, M_S_ = Molar mass of phenylalanine resp. tryptophan.
