# Supplementary material for: Modified Bacterial Cellulose Dressings to Treat Inflammatory Wounds
Source: Nanomaterials (Basel). 2020 Dec 14;10(12):2508. doi: 10.3390/nano10122508 (PMC7764978; doi:10.3390/nano10122508)
Supplement: Supplementary file 1 [file nanomaterials-10-02508-s001.pdf]

nanomaterials

Supplementary Materials

Modified Bacterial Cellulose Dressings to Treat Inflammatory Wounds

Uwe Beekmann [1], Paul Zahel [1], Berit Karl [1], Lisa Schmölz [2,3], Friedemann Börner [4], Jana Gerstmeier [4], Oliver Werz [4,5], Stefan Lorkowski [2,3,5], Cornelia Wiegand [6], Dagmar Fischer [1,5] and Dana Kralisch [1,5,*]

[1] Pharmaceutical Technology and Biopharmacy, Institute of Pharmacy, Friedrich Schiller University, Lessingstraße 8, 07743 Jena, Germany; uwe.beekmann@uni-jena.de (U.B.); paul.zahel@gmail.com (P.Z.); berit.karl@uni-jena.de (B.K.); dagmar.fischer@uni-jena.de (D.F.); dana.kralisch@uni-jena.de (D.K.)
[2] Nutritional Biochemistry and Physiology, Institute of Nutritional Sciences, Friedrich Schiller University, Dornburger Straße 25, 07743 Jena, Germany; lisa.schmoelz@uni-jena.de (L.S.); stefan.lorkowski@uni-jena.de (S.L.)
[3] Competence Cluster for Nutrition and Cardiovascular Health (nutriCARD) Halle-Jena-Leipzig, Friedrich Schiller University, Dornburger Straße 25, 07743 Jena, Germany
[4] Pharmaceutical and Medicinal Chemistry, Institute of Pharmacy, Friedrich Schiller University, Philosophenweg 14, 07743 Jena, Germany; friedemann.boerner@uni-jena.de (F.B.); jana.gerstmeier@uni-jena.de (J.G.); oliver.werz@uni-jena.de (O.W.)
[5] Jena Center for Soft Matter (JCSM), Friedrich Schiller University, Philosophenweg 7, 07743 Jena, Germany
[6] Department of Dermatology, Jena University Hospital, Erfurter Str. 35, 07743 Jena, Germany; c.wiegand@med.uni-jena.de (C.W.)
* Correspondence: dana.kralisch@uni-jena.de

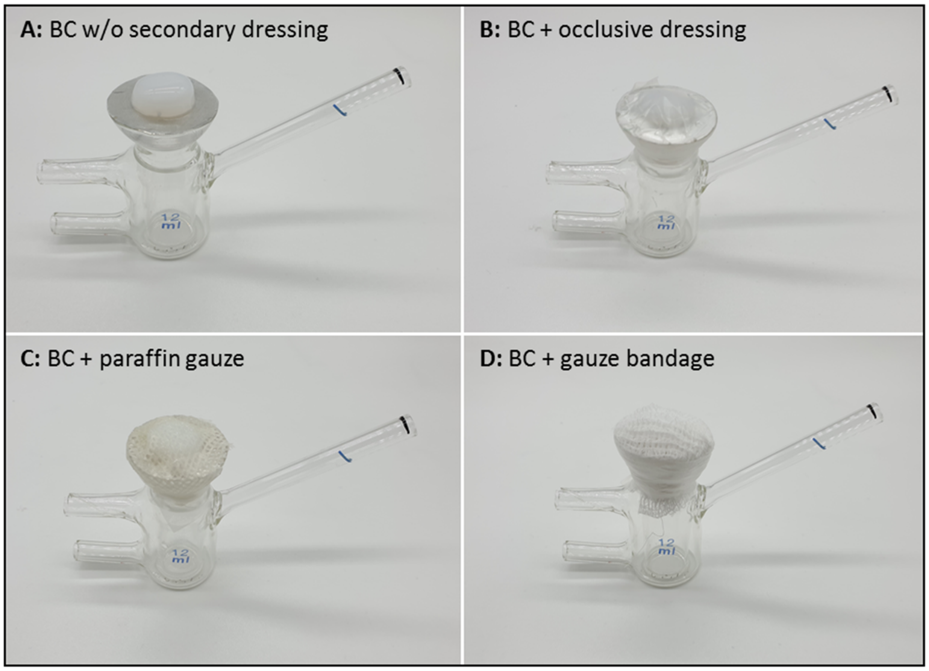

Figure S1. Fixation of indomethacin-loaded native BC samples with secondary dressings: **A:** without secondary dressing, **B**: paraffin gauze Jelonet®, **C:** occlusive dressing Opsite® Flexifix, and **D:** gauze bandage Ypsiflex® onto Franz cells. Release experiments with prepared cells were run in triplicate.

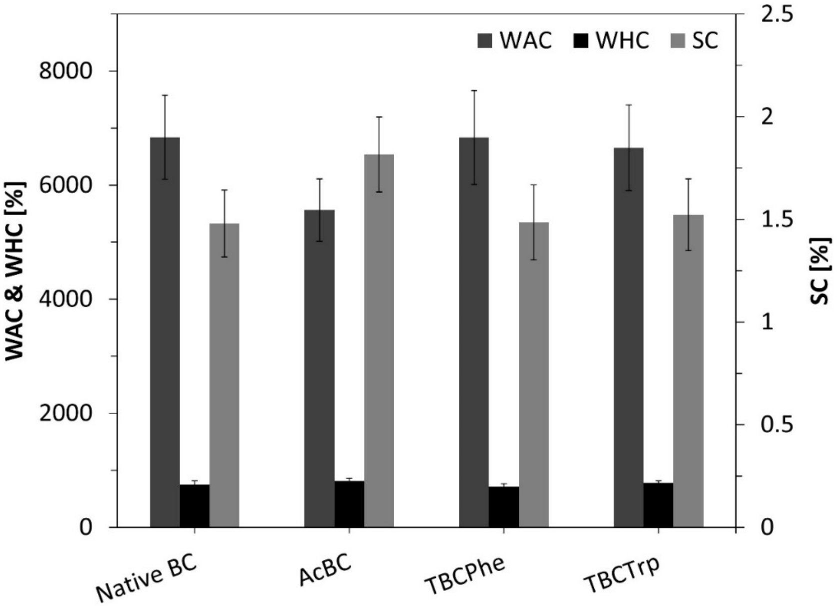

Figure S2. Water absorption capacity (WAC), water holding capacity (WHC), and solid content (SC) of native BC and *post*-modified BC samples (AcBC, TBCPhe, TBCTrp) (mean ± standard deviation; n = 8).

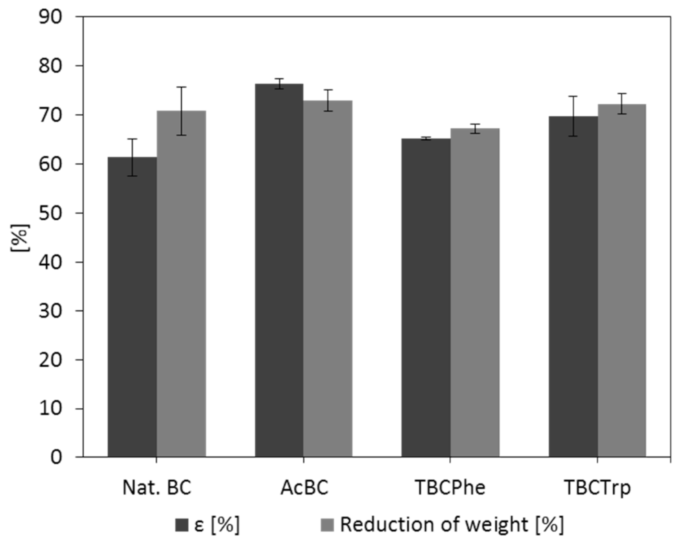

Figure S3. Reduction of weight and compressive strain (ε) of native BC compared to *post*-modified BC. Samples were burdened in perpendicular direction for ten minutes at 32 °C using a test weight of 400 g. Results are expressed as mean of three BC fleeces ± standard deviation.

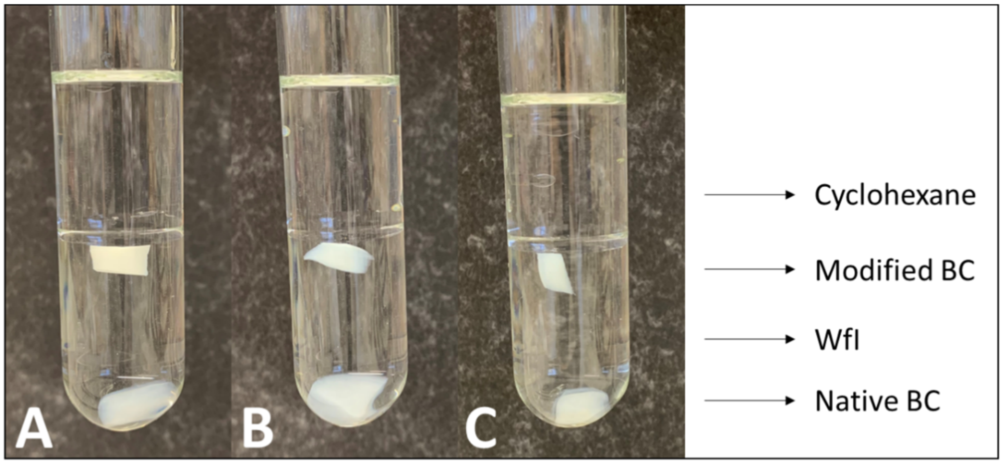

Figure S4. Qualitative hydrophobicity assay exemplarily depicted for native (**A**, **B**, **C**) and modified BC. **A:** acetylated BC (AcBC), **B**: TEMPO-oxidized BC coupled with phenylalanine (TBCPhe), **C**: TEMPO-oxidized BC coupled with tryptophan (TBCTrp)) samples. The distribution of the samples in test tubes containing two immiscible polar/nonpolar liquid phases (water/cyclohexane) was evaluated according to previous studies (Ávila Ramírez et al., 2014). The water phase is on the bottom side of the cyclohexane phase.

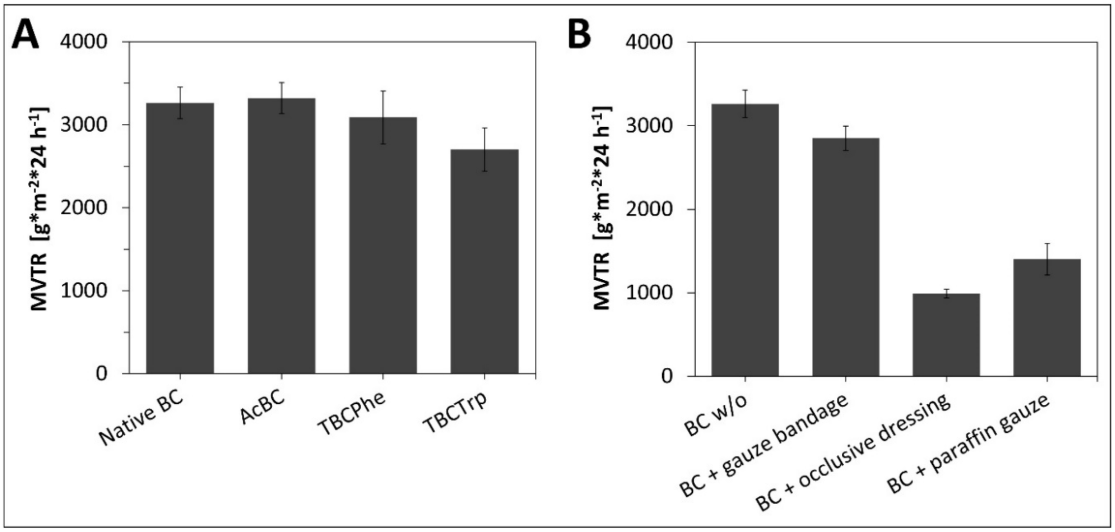

Figure S5. A: Moisture vapor transmission rate (MVTR) of BC and *post*-modified BC samples.; **B:** MVTR of native BC combined with different secondary dressing. Data are given as mean ± standard deviation (n = 5 for each group).

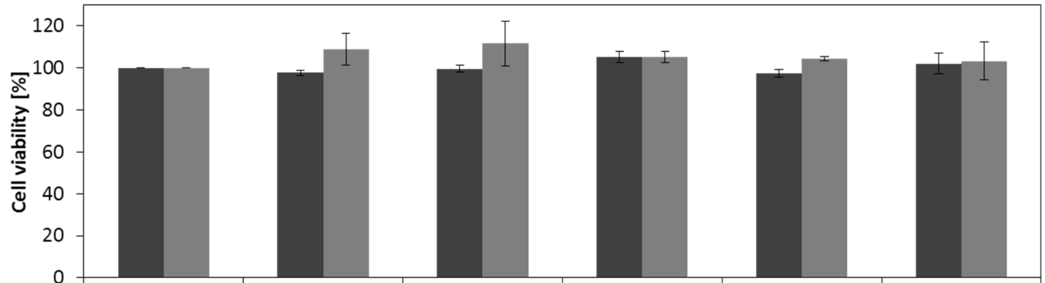

	Control	Nat. BC	AcBC	TBC	TBCPhe	TBCTrp
■ RAW264.7	100	98	100	105	97	102
■ THP-1	100	109	112	105	104	103

Figure S6. Cytotoxic effects of BC and modified BC samples on RAW264.7 cells and THP-1 cells after 24 h incubation time using 50% BC/RPMI-extracts for THP-1 cells and 50% BC/DMEM for RAW264.7 cells. The respective cell culture medium without BC extracts was used as control. Cell viability [%] was determined by MTT (3-(4,5-dimethylthiazol-2-yl)-2,5-diphenyltetrazolium bromide) assay and is shown as mean ± standard deviation of three biological replicates.

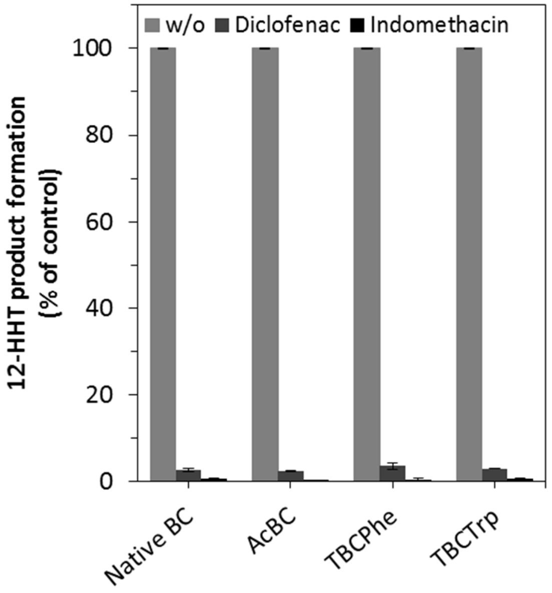

Figure S7. Inhibition of COX-1 activity (expressed as 12-hydroxyheptadecatrienoic acid (12-HHT) formation) in human platelets by released diclofenac sodium and indomethacin. Isolated platelets were incubated with the media obtained from incubation of the various BCs for five minutes. Then, 5 µM of arachidonic acid were added, and after five minutes at 37 °C, the formation of 12-HHT was determined. Data given as mean ± standard deviation; n = 3 for each group.

$$DS_{Phe} = \frac{M_{AGU} \cdot w_N}{M_N \cdot 100 - M_S \cdot w_N}$$

$$DS_{Trp} = \frac{M_{AGU} \cdot 0.5 \cdot w_N}{M_N \cdot 100 - M_S \cdot 0.5 \cdot w_N}$$

Equation S1. Equations for yield calculations of TBCPhe and TBCTrp (DS_{Phe} = Degree of substitution phenylalanine, DS_{Trp} = Degree of substitution tryptophan, M_{AGU} = Molar mass anhydroglucose unit, w_N = Mass fraction nitrogen, M_N = Molar mass nitrogen, M_S = Molar mass phenylalanine resp. tryptophan.
